# Supplementary material for: Synergistic-potential engineering enables high-efficiency graphene photodetectors for near- to mid-infrared light
Source: Nat Commun. 2024 Feb 9;15:1225. doi: 10.1038/s41467-024-45498-3 (PMC10858052; doi:10.1038/s41467-024-45498-3)
Supplement: Supplementary file 1 — Supplementary Information [file 41467_2024_45498_MOESM1_ESM.pdf]

Supplementary Information

Synergistic-potential engineering enables high-efficiency graphene photodetectors for near- to mid-infrared light

*Hao Jiang[1,2,3], Jintao Fu[1], Jingxuan Wei[4], Shaojuan Li[5], Changbin Nie[1], Feiying Sun[1], Qing Yang Steve Wu[6], Mingxiu Liu[5], Zhaogang Dong[6, *], Xingzhan Wei[1, *], Weibo Gao [2, *] and Cheng-Wei Qiu[3, *]*

[1]Chongqing Institute of Green and Intelligent Technology, Chinese Academy of Sciences, Chongqing 400714, China

[2]School of Physical and Mathematical Sciences, Nanyang Technological University, Singapore 639798, Singapore

[3]Department of Electrical and Computer Engineering, National University of Singapore, Singapore 117583, Singapore

[4]School of Optoelectronic Science and Engineering, University of Electronic Science and Technology of China, Chengdu 610054, China

[5]Changchun Institute of Optics, Fine Mechanics and Physics, Chinese Academy of Sciences, Changchun 130033, China

[6]Institute of Materials Research and Engineering (IMRE), Agency for Science, Technology and Research (A*STAR), Singapore 138634, Singapore

[*] Email:

(Z. G. D.) dongz@imre.a-star.edu.sg

(X. Z. W.) weixingzhan@cigit.ac.cn

(W. B. G.) wbgao@ntu.edu.sg

(C.W. Q.) chengwei.qiu@nus.edu.sg

Supplementary Note 1

1. Device structure and morphology characterization.

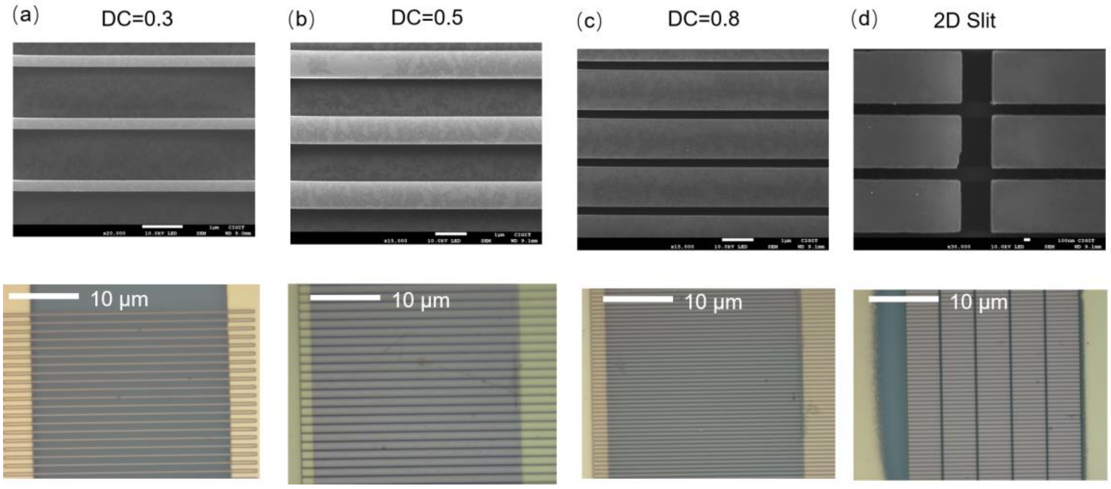

Supplementary Fig. 1. Device morphology characterization (a)-(d) SEM and optical microscope images with DC of 0.3, 0.5, 0.8 and 2D slit structures.

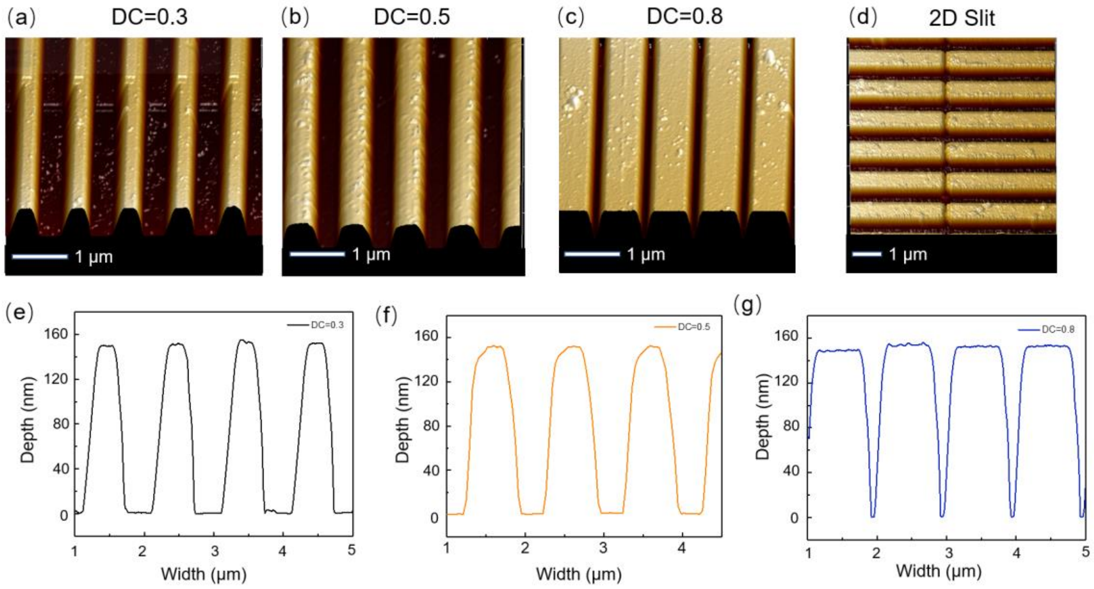

Supplementary Fig. 2. Characterization of Surface Structure Heights in the Device. (a)-(d) AFM morphology map with DC of 0.3, 0.5, 0.8 and 2D slit structures. (e)-(f) Height data with DC of 0.3, 0.5 and 0.8.

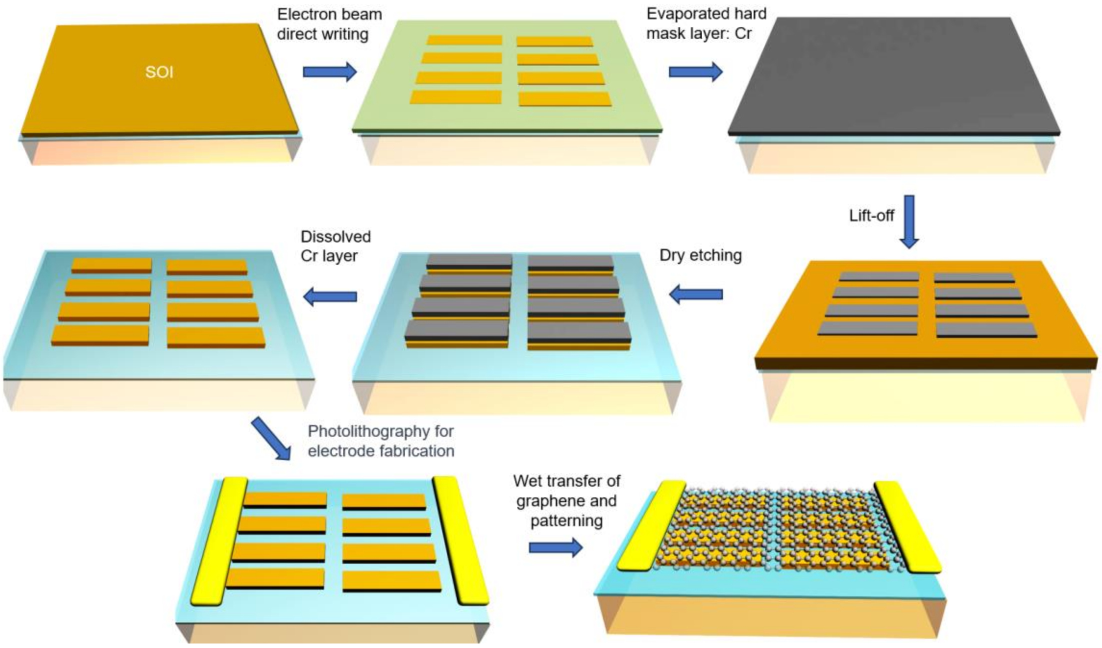

Supplementary Fig. 3. Fabrication process of devices.

Supplementary Table 1 Parameters of the graphene measured by Hall effect testing

**Parameter**	**Mobility ($cm^2/V{\cdot}s$)**	**Concentration($/cm^2$)**	**Type of dopping**	**Resistance (Ω)**
**Quantity**	8116.2	7.8×10^{11}	P	1135

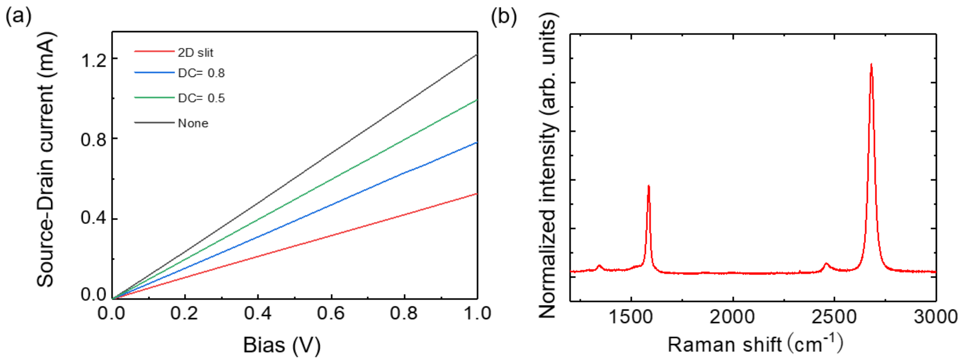

Supplementary Fig. 4. The electrical and Raman measurements of graphene devices. (a) I-V curves of devices with different structures. (b) Raman testing of graphene on silicon oxide.

Supplementary Note 2

2. Surface Electric Field Analysis and Gain Mechanism

2.1. Surface Electric Field Engineering and Gain model

The generation of electrical gain involves three main physical processes: the first is the generation of potential wells, the second is the separation of photocarriers, and the third is the process of photoconductive gain.

2.1.1 Generation of potential wells

It has been discovered that a strong electric field is located at the edge of silicon and silicon oxide, which would be stronger than the built-in electric field formed by graphene and silicon[1], which can be modeled and simulated in Supplementary Fig. 5 by Silvaco TCAD. For the slit structure, due to the electric field coupling between adjacent grating boundaries, the surface potential distribution will resemble a valley with a higher potential difference, as shown in the corresponding schematic diagram of Supplementary Fig. 5c. This potential distribution caused by the slit structure can be called the slit effect. Furthermore, when a slit structure is constructed in two mutually perpendicular directions in a two-dimensional plane, the valley potential distribution will evolve into a 2D potential well.

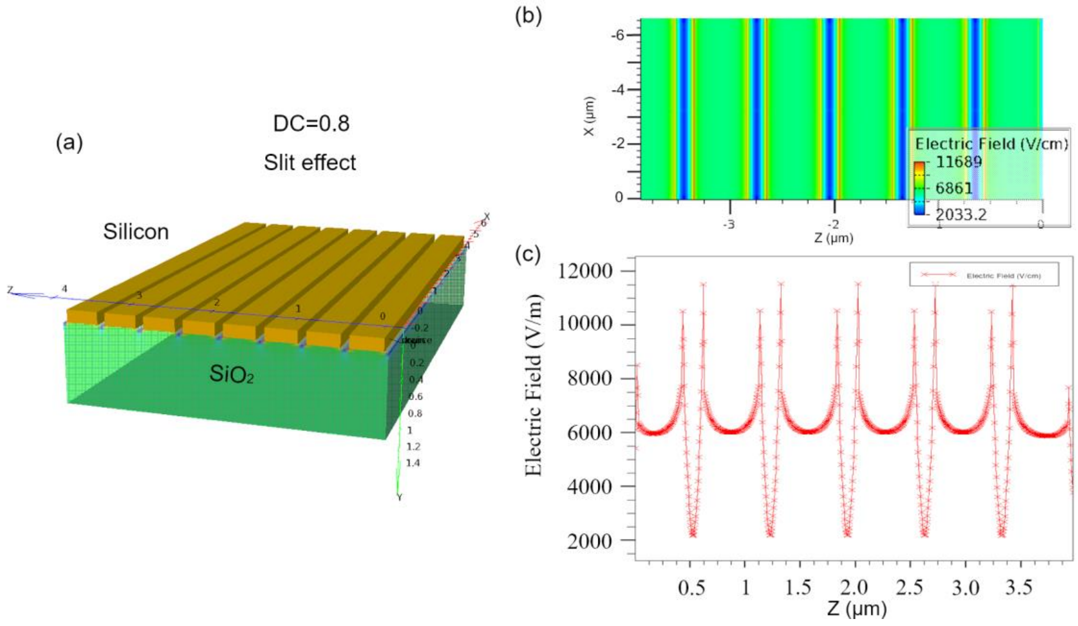

Supplementary Fig. 5. Surface potential simulation. (a) 3D structural modeling. **(b)** Surface electric field distribution map. **(c)** The relationship between surface electric field and z-position.

As a verification, we measured the surface potential of the structure based on AFM characterization and obtained the electric field distribution maps that were consistent with the simulation, as shown in Supplementary Fig. 6.

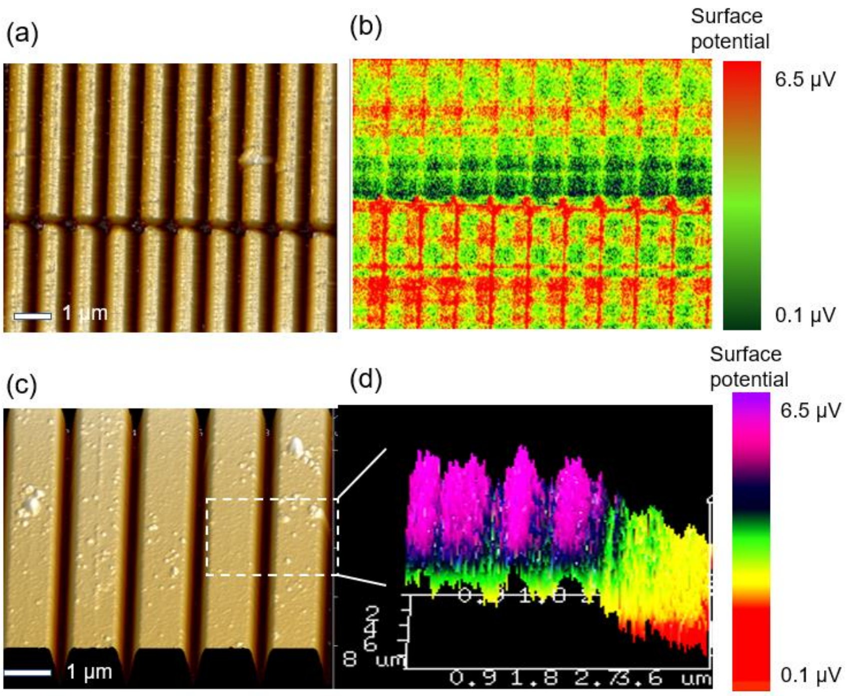

Supplementary Fig. 6. Surface potential characterization. (a)-(b) AFM image of 2D slit structure and corresponding surface potential distribution characterization diagram. **(c)-(d)** 3D AFM image of slit structure and corresponding surface potential distribution characterization diagram

2.1.2 Separation of photocarriers

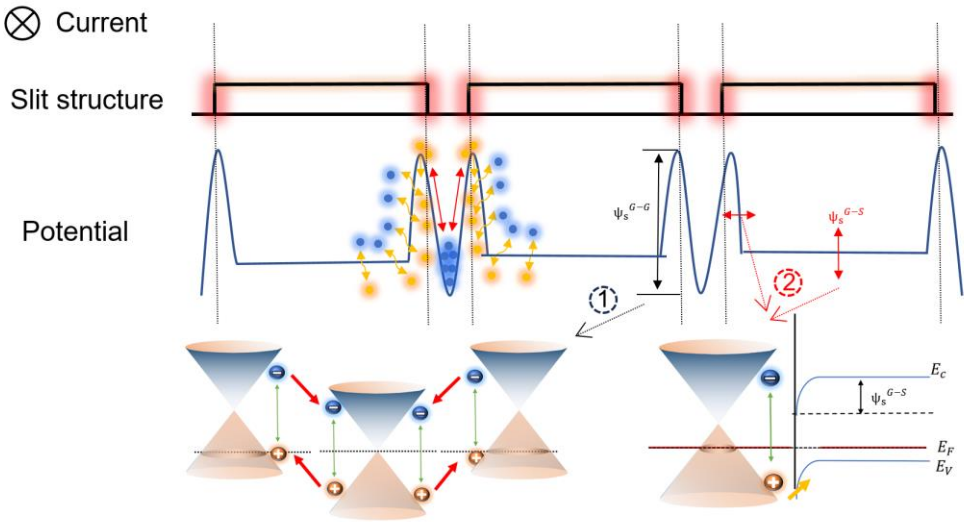

Supplementary Fig. 7. Schematic diagram of surface electric field distribution and mechanism of photo generated carrier separation.

Aforementioned fluctuation potential distribution can significantly influences the separation of photocarriers. Since this work primarily focuses on the testing of the infrared spectrum, the generation of photocarriers relies on graphene. The separation of photocarriers mainly occurs through the action of the transverse homojunction electric field of graphene for process①, as well as the action of the interface electric field between graphene and silicon as process ②, as shown in Supplementary Fig. 7.

The relationship between the energy band of the silicon/graphene interface and the position (z) is obtained by solving the Poisson equation[2]:

$$\varphi(z) = \begin{cases} \varphi(-w) & z < -w \\ \varphi(-w) - \frac{qN(z+w)^2}{2\epsilon_s} & -w < z < 0 \end{cases} \quad \text{(S1)}$$

where z=0 is the interface between silicon and graphene, w is the width of depletion zone at the interface, $N = N_D - N_A$ is the net carrier concentration of silicon, N_D is the donor impurity concentration, N_A is the acceptor impurity concentration, and ϵ_s is the dielectric constant of silicon.

The corresponding expression of the contact potential can be obtained by:

$$\psi_s^{G-S} = -q\big(\varphi(0) - \varphi(-w)\big) = \frac{qNw^2}{2\epsilon_s} \quad \text{(S2)}$$

Since graphene will not have large Fermi level shift when contacting with silicon oxide, the electric field of homojunction ψ_s^{G-G} is equal to the edge strong electric field.

Next, under the action of electric potential ψ_s^{G-G} and ψ_s^{G-S}, the photocarriers in graphene are separated and form photocurrent. For convenience, we will refer to the two sides with potential differences as the P region and N region, respectively. For the carrier distribution in the heterojunction region, the relationship between carrier concentration of P-region and quasi Fermi level is

$$n_p = n_i exp\,(\frac{E_{Fn}-E_i}{k_0T}) \quad \text{(S3)}$$

$$p_p = n_i exp\,(\frac{E_i-E_{Fp}}{k_0T}) \quad \text{(S4)}$$

Thus

$$n_p p_p = n_i^2 exp\,(\frac{E_{Fn}-E_{Fp}}{k_0 T}) \tag{S5}$$

At the boundary of P region, we define $x = -x_p$,$E_{Fn} - E_{Fp} = qV$, so

$$n_p(-x_p)p_p(-x_p) = n_i^2 exp\,(\frac{E_{Fn}-E_{Fp}}{k_0 T}) \tag{S6}$$

Because $p_p(-x_p)$ is the majority carrier in the P region, so $p_p(-x_p) = p_{p0}$, $n_{p0}p_{p0} = n_i^2$, Thus, at the boundary of P region $x = -x_p$, minority carrier concentration at P region is

$$n_p(-x_p) = n_{p0}\, exp\left(\frac{qV}{k_0 T}\right) = n_{p0} exp\,(\frac{qV - V_D}{k_0 T}) \tag{S7}$$

Thus, the photo-generated minority carrier concentration injected into the P region is obtained

$$\Delta n_p(-x_p) = n_p(-x_p) - n_{p0} = n_{p0}\left[exp\left(\frac{qV}{k_0 T}\right) - 1\right] \tag{S8}$$

Similarly, the photo-generated minority carrier concentration injected into N region at the boundary $x = -x_n$ is

$$\Delta p_n(x_n) = p_n(x_n) - p_{n0} = p_{n0}[exp\left(\frac{qV}{k_0 T}\right) - 1] \tag{S9}$$

It can be seen that the photo-generated minority carrier at the boundary of the injection barrier region is a function of the applied voltage and also a boundary condition for solving the continuity equation.

In the steady state, the continuity equation of photo-generated minority carriers in the hole diffusion region is

$$D_p \frac{d^2 \Delta p_n}{dx^2} - \mu_n \varepsilon_x \frac{d\Delta p_n}{dx} - \mu_n p_n \frac{d\varepsilon_x}{dx} - \frac{p_n - p_{n0}}{\tau_p} = 0 \tag{S10}$$

In the case of small injection $\varepsilon_x = 0$

$$D_p \frac{d^2 \Delta p_n}{dx^2} - \frac{p_n - p_{n0}}{\tau_p} = 0 \tag{S11}$$

The variation of carrier concentration in P and N regions can be solved:

$$\Delta p(x) = p_n(x) - p_{n0} = p_{n0}\left[exp\left(\frac{qV}{k_0 T}\right) - 1\right] exp\,(\frac{x_n - x}{L_p}) \tag{S12}$$

$$\Delta n(x) = n_p(x) - n_{p0} = n_{p0}\left[exp\left(\frac{qV}{k_0 T}\right) - 1\right] exp\,(\frac{x_p + x}{L_n}) \tag{S13}$$

where $qV = q(V_{bi} - V_{oc}) = \varphi_{\mathrm{i}}$. $qV_{bi} = E_{Fn} - E_{Fp}$, qV_{bi} is contact potential difference or built-in potential difference due to the difference of Fermi energy levels

of junctions in the dark state, where V_{oc} comes from the photogenerated voltage

$$V_{oc} = \frac{k_0T}{q} ln\left(\frac{I_L}{I_s} - 1\right) \tag{S14}$$

where Δn (Δp) is the injection concentration of electrons (holes), n_{p0} is the intrinsic carrier concentration, μ_n (μ_p) is the mobility of electrons (holes), E is the applied electric field. g is the generation rate of excess carriers. L is the length of graphene channel. qV_D is the barrier height due to the difference of Fermi energy levels of junctions in the dark state L_n (L_p) is the diffusion length of carrier.

It is also assumed that the holes in the diffusion length L_p and the electrons in L_n can diffuse to the other side of the pn junction. Then the photogenerated current is

$$I_L = q\bar{Q}A(L_p + L_n) \tag{S15}$$

where $\bar{Q}$ is represented as the average generation rate of photo-generated carriers within the diffusion length ($L_p + L_n$) of the junction. The interface voltage of heterojunction can be derived

$$V = V_{bi} - \frac{k_0T}{q} ln\left[\frac{q\bar{Q}A(L_p+L_n)}{I_s} - 1\right] \tag{S16}$$

where I_s is the reverse saturation current, and $qV_{bi} \propto \psi_s{}^{G-S} + \psi_s{}^{G-G}$.

2.1.3 Photoconductive gain

Under the influence of the interface electric field, one type of photocarriers is separated to the potential well region, while the other type of photocarriers, under the bias effect, undergo photoconduction within the graphene channel, resulting in photocurrent generation. The potential well traps photogenerated electrons/holes, thereby extending their recombination time. As a result, multiple conduction cycles occur within the channel before the recombination of photocarriers, leading to gain. We refer to this process as the photogating effect.

According to the Gain expression based on photogating effect[3]

$$\begin{cases} I_{ph} = gALeG \\ I_{ph} = (\Delta\sigma \cdot E)A = (\Delta n\mu_n + \Delta p\mu_p)eEA \end{cases} \tag{S17}$$

The formed built-in electric field not only promotes the separation of

photogenerated carriers in graphene, but also inhibits the recombination of photogenerated carriers, which can induce cyclic gain. After derivation with S16, the gain can be expressed as:

$$G = \frac{\Delta n(\mu_n+\mu_p)E}{gL} = \frac{n_{p0}\left[exp\left(\frac{qV}{k_0T}\right)-1\right]exp\,(\frac{x}{Ln})(\mu_n+\mu_p)E}{gL} \quad \text{(S18)}$$

where $V = V_{bi} - \frac{k_0T}{q} ln\,[\frac{q\bar{Q}A(L_p+L_n)}{I_s} - 1]$

When there is no applied voltage on the junction, $G \propto V_{bi}$. Thus, the enhancement of the built-in potential can lead to the increase of the gain. Here, the separation driving force of photo-generated carriers is provided by the surface lateral electric field and the vertical heterojunction built-in electric field. Therefore, $qV_{bi} \propto \psi_s{}^{G-S} + \psi_s{}^{G-G}$.

2.2. Photoswitching behaviors of graphene/2D slit structure photodetector upon different wavelength

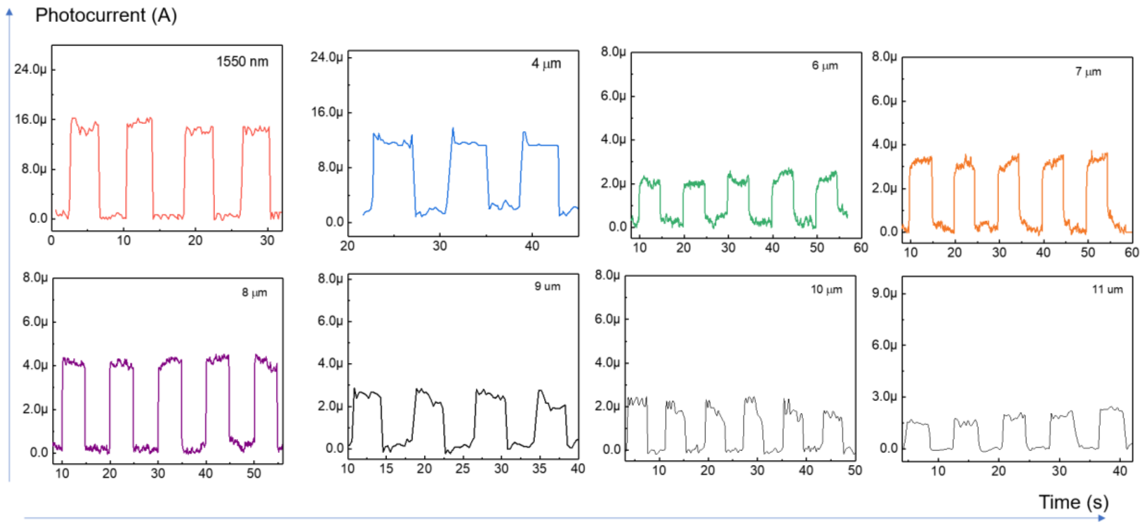

Supplementary Fig. 8. Photoswitching behaviors of graphene/2D slit structure photodetector upon different wavelength of 1550 nm, 4 μm, 6 μm, 7 μm, 8 μm, 9 μm, 10 μm and 11 μm illumination that is chopped on/off.

2.3. Validation of Potential Engineering

To verify the improvement of the responsivity of the above device is attributed to the reconstruction and enhancement of the interface potential by slit structure, we need to clarify whether the interface electric field can effectively affect the photogating mechanism for such structure devices. Since the application of back gate voltage can manipulate the carrier concentration distribution and the built-in electric field at interface region, so as to enhance the photogating effect, we can try to verify this mechanism by applying gate-modulation. Supplementary Fig. 9(a) displays the simulated interface carrier distribution of the device. When no gate voltage is applied, the photogenerated carriers are separated under the built-in field of the graphene/silicon heterojunction, with one type of carrier injected into the graphene and the other type of carrier trapped in the silicon, as shown in the top-figure. When a back-gate voltage is applied, carrier accumulation effect is formed in the semiconductor material, leading the injection effect into graphene. as shown in the bottom-figure. This can increase the built-in electric field at the interface, thereby enhancing the efficiency of photogenerated carrier separation. The corresponding energy band diagram is shown in Supplementary Fig. 9(b). The carrier injection effect caused by gate voltage can effectively enhance the interface electric field by changing the Fermi level of graphene. Applying gate voltage modulation to devices with DC of 0.3 and 0.8 caused a consistent improvement in response signal to light at 1.55 μm wavelength (Supplementary Fig. 9(c)-(d)). This indicates that both the slit structure and back-gate voltage can improve the device responsivity by enhancing the interface electric field. However, the electric field enhancement caused by gate voltage is planar uniform and has weaker ability to manipulate the Fermi level of graphene. The slit structure can achieve two-dimensional enhancement of both interface heterojunction electric field and homojunction electric field, and the enhancement effect is greater than the former. In addition, the dielectric structure without gate voltage provides more design flexibility.

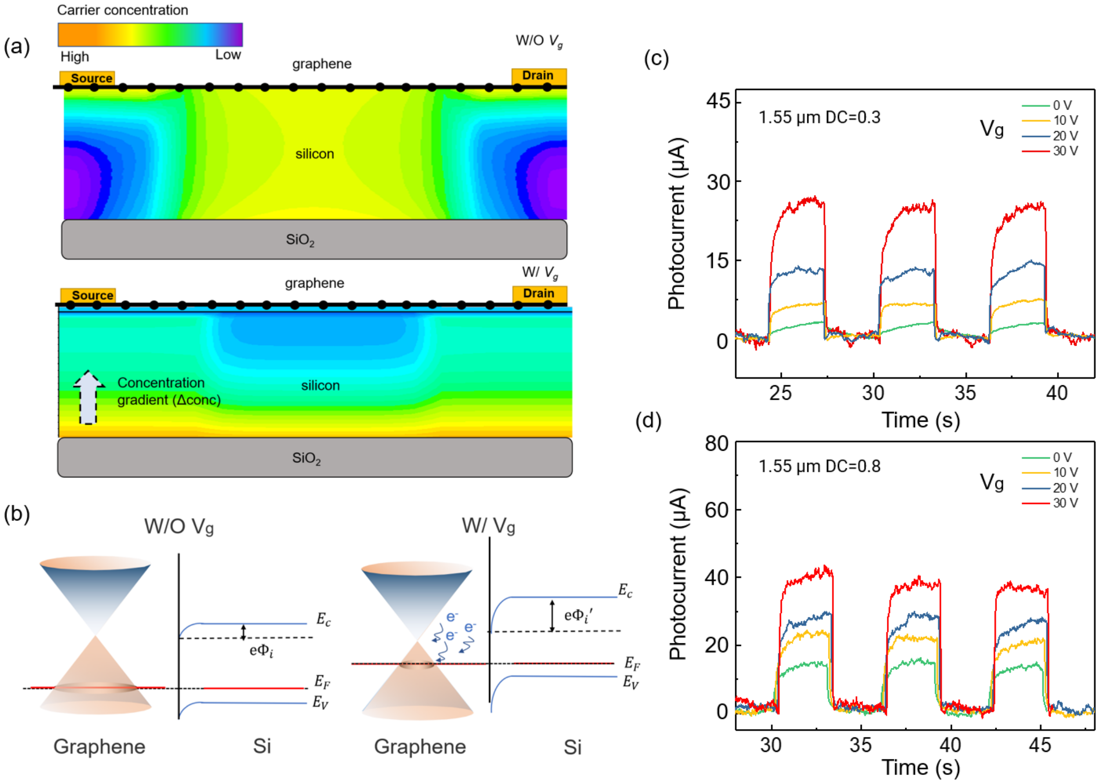

Supplementary Fig. 9. Analysis of gate voltage characteristics of the device. (a) Schematic diagram of the manipulation of gate voltage on carrier distribution. The figure above is the simulated carrier distribution without gate voltage, the separation of photogenerated carriers depends on the intrinsic potential of the interface. The figure below shows the simulated carrier distribution under gate voltage. The formation of enhanced potential and concentration gradients will promote the separation of photogenerated carriers. (b) The energy band diagram of the graphene/silicon interface with and without applying the gate voltage. (c)-(d) The enhancement effect of gate voltage on the photoelectric response of devices with DC=0.3 and 0.8, respectively. The wavelength of the incident light is 1.55 μm.

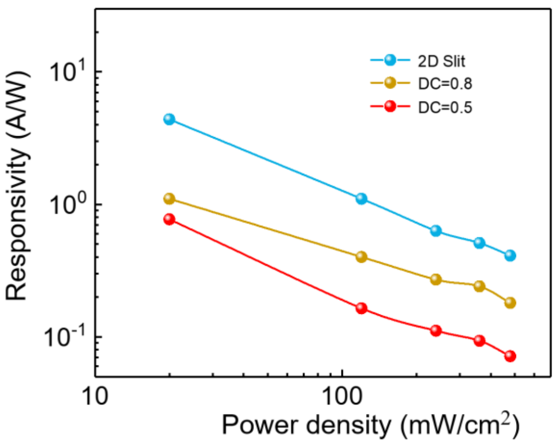

Supplementary Fig. 10. The relationship between device responsivity and 1.55 μm laser power density

Supplementary Note 3

3. Research on Polarization Sensitive Detection of Device.

3.1. Polarization sensitive design by silicon gratings

Throughout the entire process of photodetection, the first step involves the incidence and absorption of light, while the second step involves the conversion of light into an electrical signal (mentioned above). Therefore, for polarization-sensitive detection, this is achieved through the polarization ratio of incident and absorbed light. Specific-sized grating structures have the ability to induce different local and reflective properties for light polarized in different directions, which we refer to as artificial anisotropy. Here, silicon gratings exhibit different resonant effects on polarized light parallel and perpendicular to the grating direction. Therefore, by adjusting the duty cycle of the silicon grating, polarization sensitive detection can be achieved by localizing polarized light of different wavelength. For example, during the process of adjusting the duty cycle from 0.3 to 0.4, the peak of the reflection spectrum exhibits a red shift, as shown in Supplementary Fig. 11. The polarized light reflection spectrum measured by FTIR is highly consistent with the simulation results. Near Field Distribution of DC=0.3 Grating structure at different polarization angles are shown in Supplementary Fig. 13.

Accordingly, by design, the device exhibits polarization-sensitive detection for polarized light with a wavelength of 4 μm when the duty cycle is 0.8, as shown in Supplementary Fig. 12.

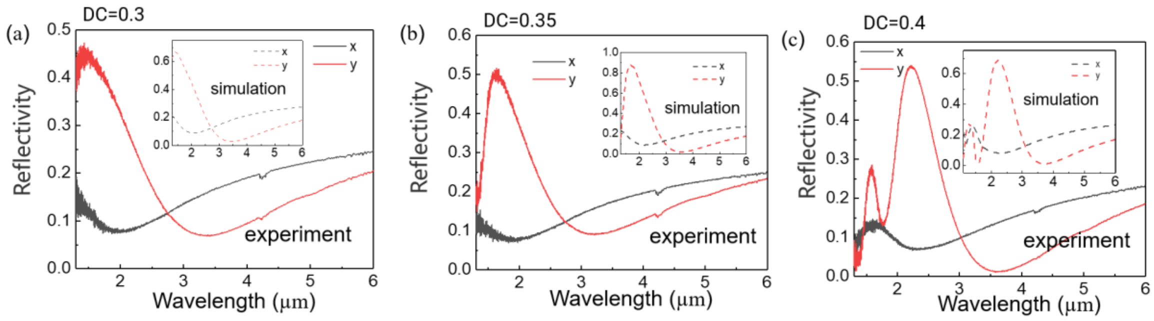

Supplementary Fig. 11. Analysis of the reflection characteristics of the grating structure. (a)-(c) The polarization sensitive reflection spectra of structures with DC of 0.3, 0.35, and 0.4 measured by FTIR. The illustration shows the corresponding far-field simulation results. The grating height is 160 nm, the unit length is 1 μm

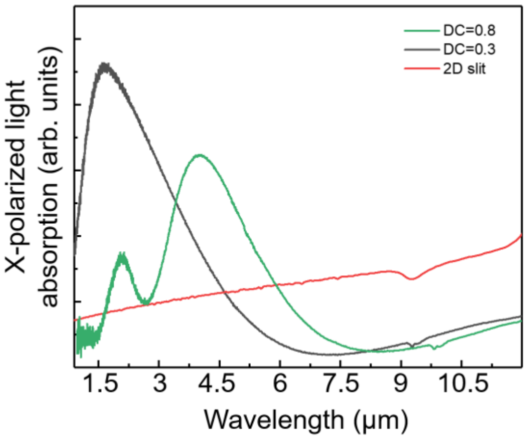

Supplementary Fig. 12. X-polarized absorption spectra of devices with different dielectric structures

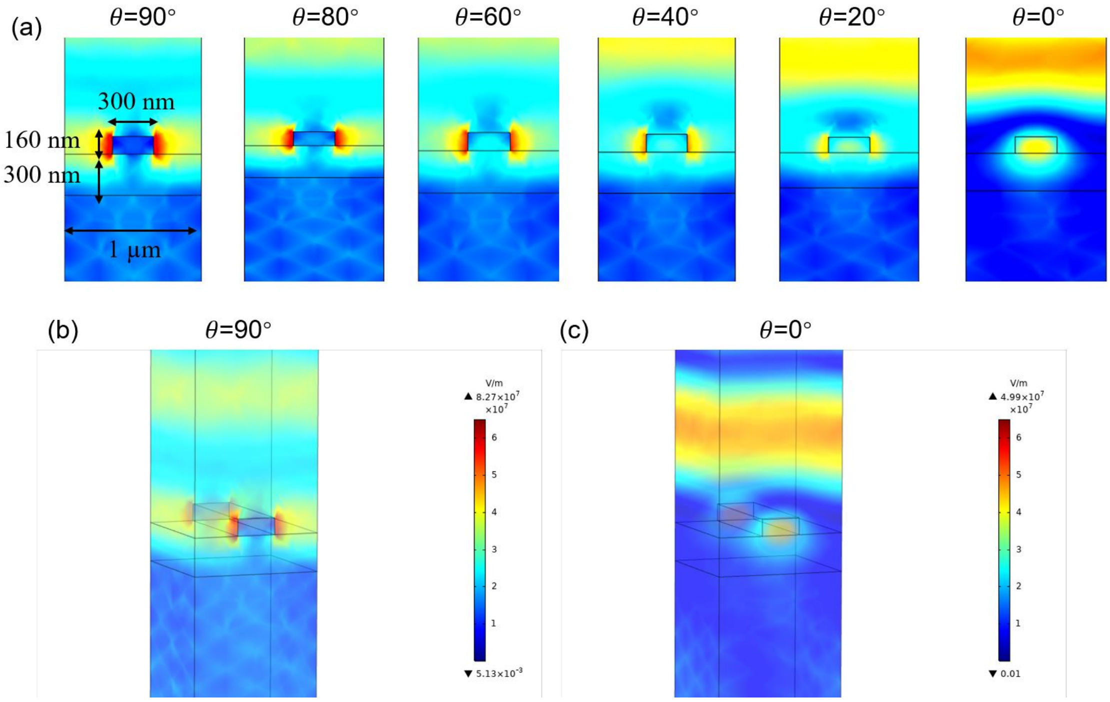

Supplementary Fig. 13. Near field simulation of grating structures. (a) Near field distribution of DC=0.3 Grating Structure at Different Polarization Angles (θ). λ=1550 nm. (b)-(c) Distribution of three-dimensional structures with θ of 90 ° and 0 °, λ=1550 nm.

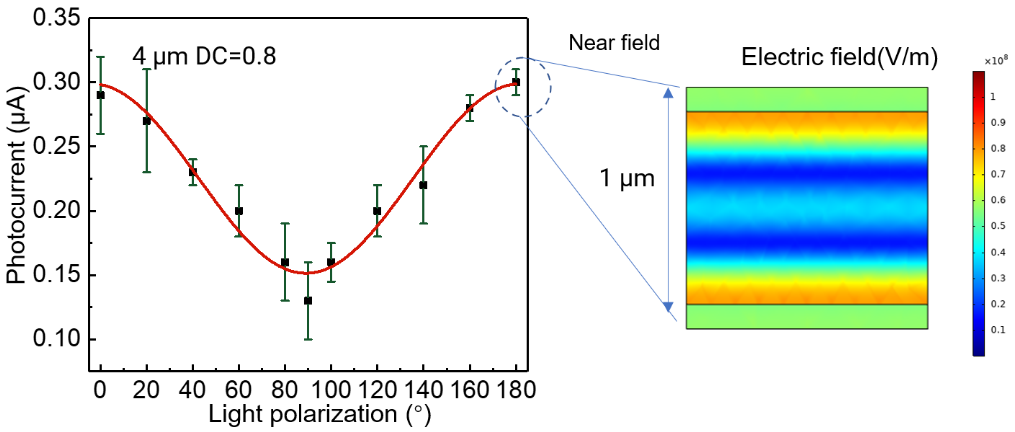

Supplementary Fig. 14. The relationship between the magnitude of the photocurrent measured multiple times and the polarization angle of the incident light at 4 μm. DC=0.8. The figure right shows the near-field characteristics corresponding to Polarization angle=0° or 180°. The grating height is 160 nm, the unit length is 1 μm

3.2. The influence of graphene fermi level on polarization ratio

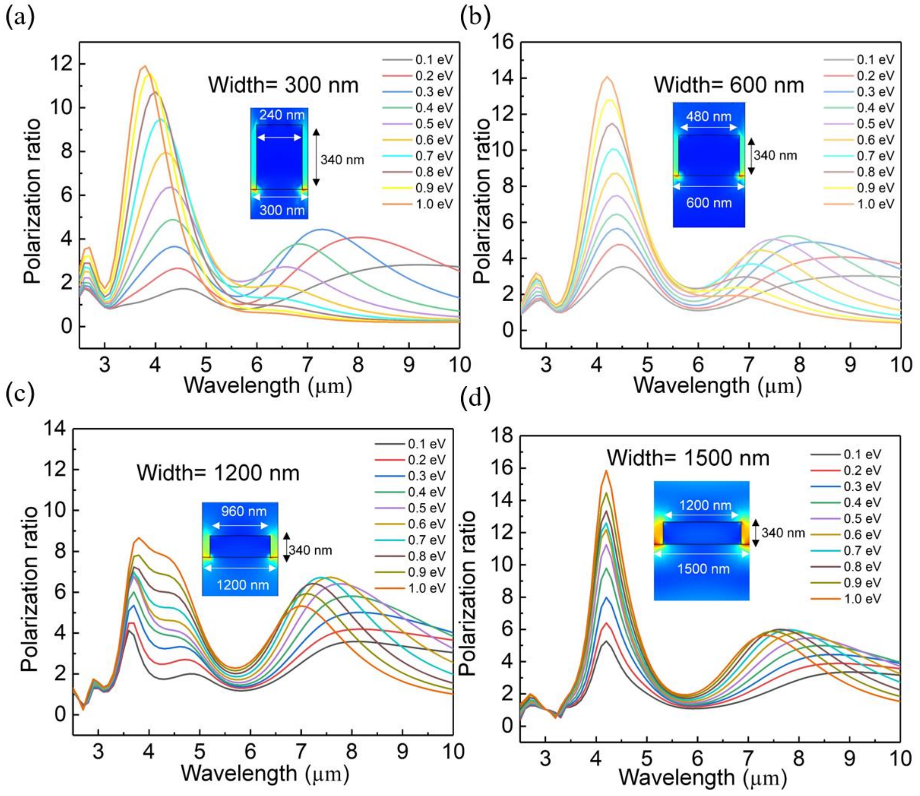

Supplementary Fig. 15. Simulation analysis of the device absorption spectrum based on graphene Fermi level. (a)-(d) The relationship between Fermi level and polarization ratio under different structure designs.

In addition to the above polarization sensitive detection method through structural design, the change of graphene Fermi level can optimize the polarization ratio. When the thickness of the grating increases to 340 nm, a large reflection polarization ratio cannot be observed in the infrared band. That is to say, relying solely on the structure cannot achieve polarization sensitive detection. The optical absorption of doped graphene is composed of intraband and interband transitions of electrons, and the approximate expression for its conductivity can be written as follows[4]：

$$\sigma(\omega)=\frac{2e^2k_BT}{\pi\hbar^2}\frac{i}{\omega+i\tau^{-1}}\ln[2\cosh(\frac{E_F}{2k_BT})] \\ +\frac{e^2}{4\hbar}[\frac{1}{2}+\frac{1}{\pi}\arctan(\frac{\hbar\omega-2E_F}{2k_BT})-\frac{i}{2\pi}\ln\frac{(\hbar+2E_F)^2}{(\hbar\omega-2E_F)^2+(2k_BT)^2}] \quad \text{(S19)}$$

where $\hbar\approx 6.63\times10^{-34}$ J·s is planck constant, $\tau=\mu E_F/(ev_F{}^2)$ is the relaxation time，μ is

carrier mobility， e is elementary charge, $v_F \approx 10^6$ m/s is Fermi velocity， k_B is the Boltzmann constant. When the photon energy is less than twice the Fermi level, the intraband transition of electrons in graphene dominates the absorption process. Therefore, for the infrared band, $E_F \gg k_B T$. Equation (19) can be simplified to the following Drude form [5]：

$$\sigma_D = \frac{ie^2 E_F}{\pi\hbar^2(\omega + i\tau^{-1})} \tag{S20}$$

By adjusting the Fermi level of graphene, the conductivity can be changed accordingly. Furthermore, the plasmon of graphene is regulated to achieve a high polarization ratio.[6] The relationship between Fermi level and absorption polarization ratio under different structure designs is shown in Supplementary Fig. 15. This provides an additional dimension for the design of polarization sensitive detection structures.

Supplementary Note 4

4. Black-body radiation and noise

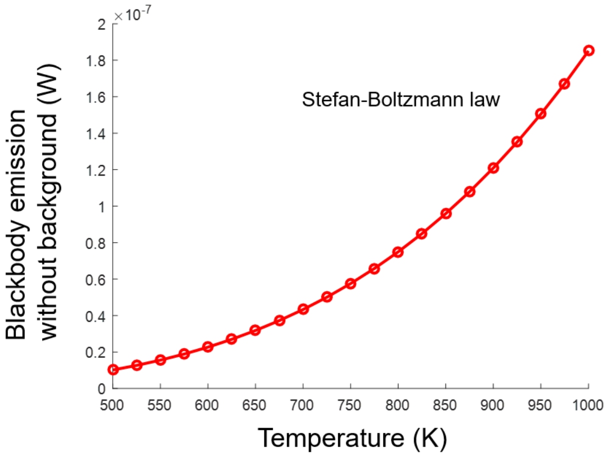

Supplementary Fig. 16. The relationship between the real radiation power and the blackbody temperature considering factors of radiation distance, spot size, device size, and background radiation.

Here, the calculation of real black-body radiation power on device P needs to take into account the blackbody temperature T, background temperature T_0, aperture radius of the blackbody radiation source r, the distance between the aperture and the detector d, the detector photosensitive area A and black-body radiation rate E_r. According to Stefan-Boltzmann law:[7-9]

$$E_r = \frac{2\pi^5 k_B^4}{15h^3c^2} \cdot (T^4 - T_0^4) \tag{S21}$$

where c is the speed of light, h is the Planck constant, and k_B is the Boltzmann constant. Here, T_0= 300 K, d =7 cm, r=6.35 mm, A=20 μm×40 μm. Thus P can be caculated by:

$$P = \frac{E_r \cdot r^2 \cdot A}{d^2} \tag{S22}$$

The responsivity under Black-body radiation can be obtained by:

$$R_p = \frac{I_p}{P} \tag{S23}$$

where I_p is the measured photocurrent. Furthermore, the relationship between equivalent noise power (NEP) with temperature under blackbody radiation was obtained by:[10]

$$NEP = \frac{\mathrm{i_n}}{R_p} = \frac{\sqrt{S(f_n)}}{R_p} \tag{S24}$$

where $S(f_n)$ is the real-time measured current noise power spectral density, f_n~ 1kHz is the center frequency of the device. The detectivity of 1.88×10^{9} cm $Hz^{1/2}$ W^{-1} can be obtained by:

$$D^* = \frac{\sqrt{A \cdot \Delta f}}{NEP} \tag{S25}$$

where Δf represents the working bandwidth (1 Hz), and A is the device area. According to the above measurement methods, we tested and calculated devices with different structures, and the results and specific indicators are shown in the Supplementary Fig. 18 and Supplementary Table 2.

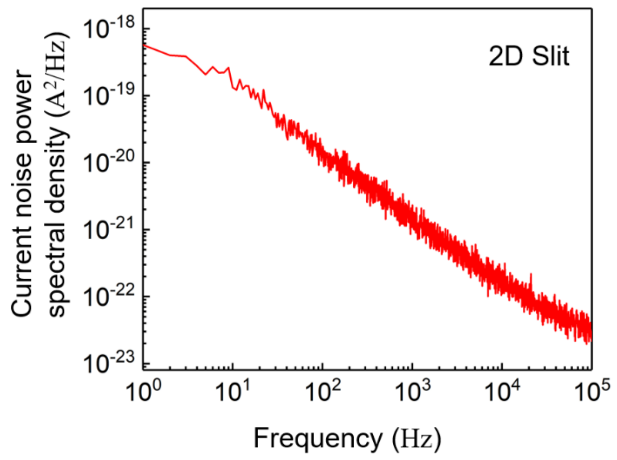

Supplementary Fig. 17. Measured current noise power spectral density of graphene/2D slit structure photodetector.

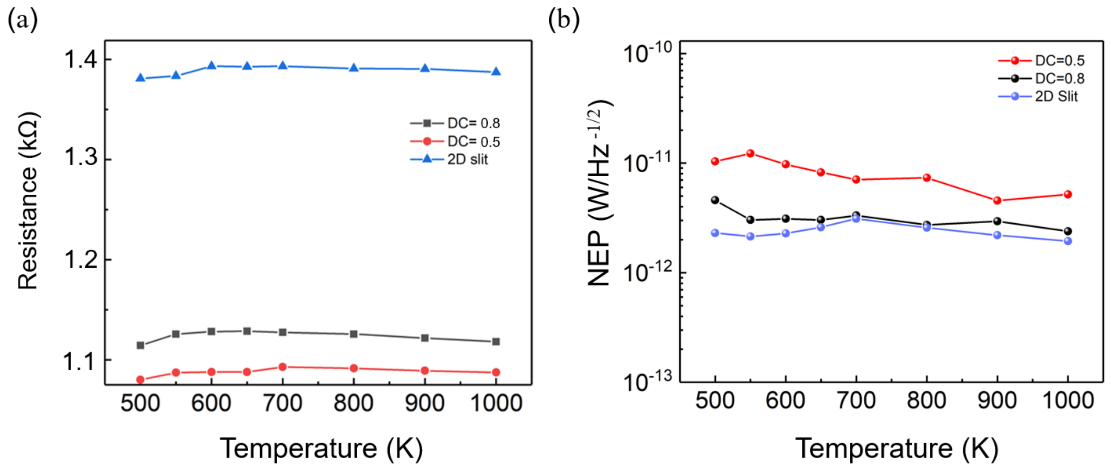

Supplementary Fig. 18. The relationship between the resistance (a) and NEP (b) of devices with different configuration structures as a function of blackbody temperature.

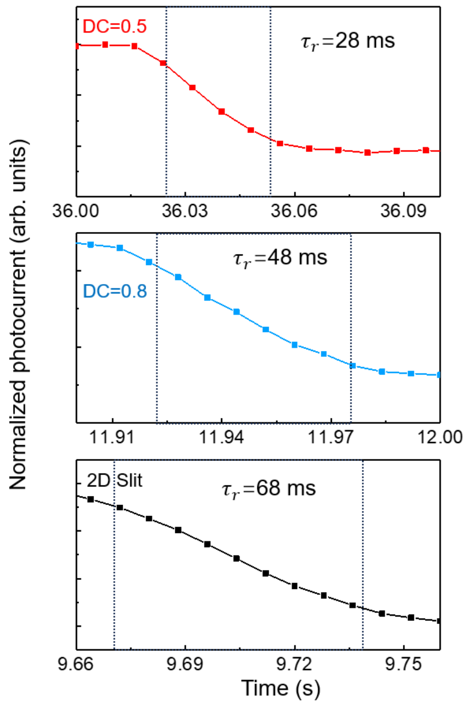

Supplementary Fig. 19. Falling edge time of devices with different dielectric structures under 1000 K blackbody radiation.

Supplementary Table 2. Noise current (I_n), equivalent noise power (NEP), responsivity and specific detection rate (D*) of devices with different structures under black-body radiation.

	T (K)	500	550	600	650	700	800	900	1000
DC=0.5	I_n (A/Hz$^{1/2}$)	4.6×10^{-11}	6.2×10^{-11}	5.2×10^{-11}	5.2×10^{-11}	4.6×10^{-11}	5.1×10^{-11}	4.5×10^{-11}	6.2×10^{-11}
	Responsivity (A/W)	4.43	5.07	5.29	6.29	6.47	6.87	9.92	11.99
	NEP (W/Hz$^{-1/2}$)	1.0×10^{-11}	1.2×10^{-11}	9.7×10^{-12}	8.2×10^{-12}	7.1×10^{-12}	7.4×10^{-12}	4.5×10^{-12}	5.2×10^{-12}
	D*(Jones)	1.9×10^{8}	1.6×10^{8}	2.1×10^{8}	2.4×10^{8}	2.8×10^{8}	2.7×10^{8}	4.4×10^{8}	3.9×10^{8}
DC=0.8	I_n (A/Hz$^{1/2}$)	5.0×10^{-11}	3.3×10^{-11}	3.4×10^{-11}	3.5×10^{-11}	4.0×10^{-11}	3.6×10^{-11}	4.0×10^{-11}	4.3×10^{-11}
	Responsivity (A/W)	10.8	10.9	11.0	11.6	12.2	13.3	13.5	18.0
	NEP (W/Hz$^{-1/2}$)	4.6×10^{-12}	3.0×10^{-12}	3.1×10^{-12}	3.0×10^{-12}	3.3×10^{-12}	2.7×10^{-12}	3.0×10^{-12}	2.4×10^{-12}
	D*(Jones)	4.4×10^{8}	6.6×10^{8}	6.4×10^{8}	6.6×10^{8}	6.0×10^{8}	7.3×10^{8}	6.8×10^{8}	8.4×10^{8}
2D slit	I_n (A/Hz$^{1/2}$)	4.8×10^{-11}	4.5×10^{-11}	5.0×10^{-11}	5.2×10^{-11}	6.5×10^{-11}	5.5×10^{-11}	5.4×10^{-11}	5.2×10^{-11}
	Responsivity (A/W)	20.7	20.9	22.0	20	20.8	21.4	24.5	27.0
	NEP (W/Hz$^{-1/2}$)	2.3×10^{-12}	2.1×10^{-12}	2.3×10^{-12}	2.6×10^{-12}	3.1×10^{-12}	2.6×10^{-12}	2.2×10^{-12}	1.9×10^{-12}
	D*(Jones)	8.7×10^{8}	9.4×10^{8}	8.8×10^{8}	7.7×10^{8}	6.4×10^{8}	7.8×10^{8}	9.1×10^{8}	1.0×10^{9}

Supplementary Note 5

5. Comparison of performance indicators.

Supplementary Table 3. Summary of device parameters of several typical graphene/semiconductor photodetectors previously reported, and our own device.

Types of devices	Working mechanism	Responsivity	Wavelength
Graphene/Si [11]	Photodiode	0.28 A/W	1550 nm
TPA-doped tri-layer graphene/Si [12]	Photodiode	0.435 A/W	800 nm
Graphene/CNT/SiO_2/Si [15]	Photodiode	0.21 A/W	980 nm
Graphene–silicon-on-insulator [16]	Photodiode	0.029 A/W	980 nm
Graphene double-layer heterostructure [18]	photo-thermoelectric	1.1 A/W	3200 nm
Graphene–silicon heterojunction in conductor mode [13]	Photoconductor	0.23 A/W	1550 nm
PtNPs/graphene/Si [14]	Photoconductor	26 A/W	790 nm
$MoTe_2$/Graphene [17]	Photoconductor	60 A/W	1064 nm
Au NP array/graphene [19]	Photoconductor	83 A/W	1550nm
Graphene/ WS_2 [20]	Photoconductor	0.735 A/W	1550nm
Graphene/silicon grating [21]	Photoconductor	25 A/W	2700nm
This work	Photogating	0.2 A/W-38 A/W	1500-11000 nm

The device type in this paper is a photoconductive device based on the photogating effect, distinct from photodiodes and photo-thermoelectric devices, each of these three device types has its own advantages and disadvantages. Photodiodes and photo-thermoelectric devices utilize the separation and flow of photocarriers under asymmetric potential or temperature gradients, allowing them to operate at zero bias voltage, with low dark current and a high ON/OFF ratio. However, they lack gain and exhibit low responsivity as disadvantages. Photoconductive devices based on photogating effect primarily rely on the separation and recombination of charge carriers in vertical heterojunctions or traps to generate gain, necessitating operation under bias voltage, hence achieving high responsivity. This, however, leads to the challenge of elevated dark current, which requires addressing in future work. In this paper, the non-

uniform distribution of heterojunctions to some extent helps suppress the dark current in the graphene channel. These discussions indeed need to be summarized at the end of the paper.

In Supplementary Table 3, all these devices depend on graphene for light absorption, making photoconductive devices with gain advantageous for broad-spectrum detection. Our research harnesses the synergistic interaction between optical and electrical fields, enabling us to surpass the performance advantages documented in existing literature.

Supplementary Note 6

6. Set up of device testing system

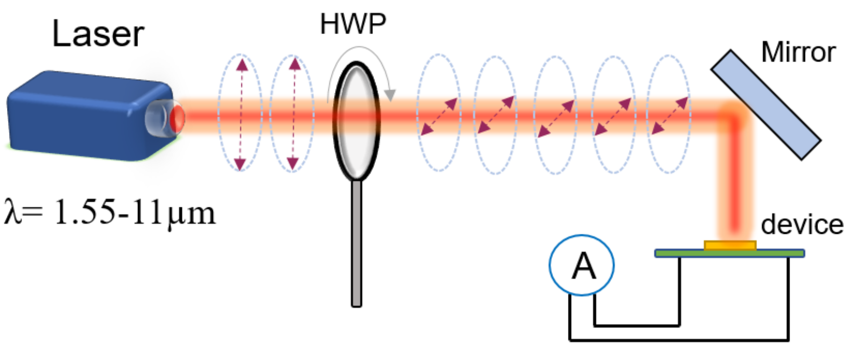

Supplementary Fig. 20. Set up of device testing system. The fabricated devices are characterized by lasers and quantum cascade lasers of different wavelengths. The polarization states of the incident light are linear, with their angles controlled via rotation of a half-wave plate. A mirror is used to deflect light rays to illuminate the sample vertically

All tests were conducted with a bias voltage of 0.1V.

Supplementary Table 4 Parameters for Responsiveness Characterization

Wavelength (μm)	Spot area (mm^2)	Power (mW)	Optical power density (mW/cm^2)	Device area (mm^2)	Photocurrent (μA)	Responsivity (A/W)
1.55	38	10	26.32	0.0016	16	38
4	50	50	100	0.0016	10	6.25
6	28	100	357.14	0.0016	4	7
7	28	100	357.14	0.0016	3	5.25
8	28	100	357.14	0.0016	2	3.5
9	28	140	500	0.0016	3	3.75
10	28	130	464.29	0.0016	2.5	3.37
11	28	120	428.57	0.0016	1.5	2.19

The responsivity of device can be obtained by:

$$R = \frac{\Delta I}{P_e} = (I_{light} - I_{dark}) \times (P \times \frac{S_{device}}{S_{light}})^{-1} \quad \text{(S26)}$$

where ΔI is photocurrent, P is optical power, S_{device} and S_{light} are the areas of active region and spot, respectively. Here, optical power density can be defined as $\frac{P}{S_{light}}$. Due to the fact that the spot area is significantly larger than the active area of the device, and the central region of the spot on device is located is far from the region of power decay at the edges, it can be approximated as a uniform distribution of optical power. Therefore, the definition of optical power density is more applicable here.

Supplementary References

1. Riazimehr, S. et al. High responsivity and quantum efficiency of graphene/silicon photodiodes achieved by interdigitating Schottky and gated regions. *Acs Photonics* **6**, 107-115 (2018).
2. Li, T. et al. Spatially controlled electrostatic doping in graphene p-i-n junction for hybrid silicon photodiode. *npj 2D Materials and Applications* **2**, 36 (2018).
3. Fang, H. & Hu, W. Photogating in Low Dimensional Photodetectors. *Advanced science* **4**, 1700323 (2017).
4. Grigorenko, A.N., Polini, M. & Novoselov, K.S. Graphene plasmonics. *Nature Photonics* **6**, 749-758 (2012).
5. Jablan, M., Buljan, H. & Soljačić, M. Plasmonics in graphene at infrared frequencies. *Physical review B* **80**, 245435 (2009).
6. Yao, W. et al. Electrically tunable graphene metamaterial with strong broadband absorption. *Nanotechnology* **32**, 075703 (2020).
7. Peng, M. et al. Room-Temperature Blackbody-Sensitive and Fast Infrared Photodetectors Based on 2D Tellurium/Graphene Van der Waals Heterojunction. *ACS Photonics* **9**, 1775-1782 (2022).
8. Wang, Y. et al. Fast Uncooled Mid-Wavelength Infrared Photodetectors with Heterostructures of van der Waals on Epitaxial HgCdTe. *Advanced materials* **34**, e2107772 (2022).
9. Peng, M. et al. Blackbody-sensitive room-temperature infrared photodetectors based on low-dimensional tellurium grown by chemical vapor deposition. *Science advances 7*, eabf7358 (2021).
10. Fang, Y.J., Armin, A., Meredith, P. & Huang, J.S. Accurate characterization of next-generation thin-film photodetectors. *Nature Photonics* **13**, 1-4 (2019).
11. Amirmazlaghani, M., Raissi, F., Habibpour, O., Vukusic, J. & Stake, J. Graphene-Si schottky IR detector. *IEEE Journal of Quantum electronics* **49**, 589-594 (2013).
12. An, X., Liu, F., Jung, Y.J. & Kar, S. Tunable graphene-silicon heterojunctions for ultrasensitive photodetection. *Nano letters* **13**, 909-916 (2013).
13. Chen, Z.F. et al. High Responsivity, Broadband, and Fast Graphene/Silicon Photodetector in Photoconductor Mode. *Advanced Optical Materials* **3**, 1207-1214 (2015).
14. Huang, K. et al. High and fast response of a graphene–silicon photodetector coupled with 2D fractal platinum nanoparticles. *Advanced Optical Materials* **6**, 1700793 (2018).
15. Zhang, T.F. et al. Broadband photodetector based on carbon nanotube thin film/single layer graphene Schottky junction. *Scientific reports* **6**, 38569 (2016).
16. Selvi, H., Hill, E.W., Parkinson, P. & Echtermeyer, T.J. Graphene-silicon-on-insulator (GSOI) Schottky diode photodetectors. *Nanoscale* **10**, 18926-18935 (2018).

17. Yu, W.Z. et al. Near-Infrared Photodetectors Based on MoTe2/Graphene Heterostructure with High Responsivity and Flexibility. *Small* **13**, 1700268 (2017).
18. Liu, C.H., Chang, Y.C., Norris, T.B. & Zhong, Z.H. Graphene photodetectors with ultra-broadband and high responsivity at room temperature. *Nature nanotechnology* **9**, 273-278 (2014).
19. Chen, X. et al. Improving the Performance of Graphene Phototransistors Using a Heterostructure as the Light-Absorbing Layer. *Nano letters* **17**, 6391-6396 (2017).
20. He, T.Y. et al. Enhanced responsivity of a graphene/Si-based heterostructure broadband photodetector by introducing a WS2 interfacial layer. *Journal of Materials Chemistry C* **9**, 3846-3853 (2021).
21. Jiang, H. et al. Enhanced Photogating Effect in Graphene Photodetectors via Potential Fluctuation Engineering. *ACS nano* **16**, 4458-4466 (2022).
